# Differential expression of mRNA isoforms in the skeletal muscle of pigs with distinct growth and fatness profiles

**DOI:** 10.1186/s12864-018-4515-2

**Published:** 2018-02-14

**Authors:** Tainã Figueiredo Cardoso, Raquel Quintanilla, Anna Castelló, Rayner González-Prendes, Marcel Amills, Ángela Cánovas

**Affiliations:** 1 Department of Animal Genetics, Centre for Research in Agricultural Genomics (CRAG), CSIC-IRTA-UAB-UB, Campus de la Universitat Autònoma de Barcelona, Bellaterra, 08193 Barcelona, Spain; 2 CAPES Foundation, Ministry of Education of Brazil, Brasilia D.F, 70.040-020 Brazil; 3 Animal Breeding and Genetics Programme, Institute for Research and Technology in Food and Agriculture (IRTA), Torre Marimon, 08140 Caldes de Montbui, Spain; 4 Departament de Ciència Animal i dels Aliments, Universitat Autònoma de Barcelona, Bellaterra, 08193 Barcelona, Spain; 5 Centre for Genetic Improvement of Livestock, Department of Animal Biosciences, University of Guelph, Guelph, ON Canada

**Keywords:** Alternative splicing, mRNA isoform, Swine, Differential expression

## Abstract

**Background:**

The identification of genes differentially expressed in the skeletal muscle of pigs displaying distinct growth and fatness profiles might contribute to identify the genetic factors that influence the phenotypic variation of such traits. So far, the majority of porcine transcriptomic studies have investigated differences in gene expression at a global scale rather than at the mRNA isoform level. In the current work, we have investigated the differential expression of mRNA isoforms in the *gluteus medius* (GM) muscle of 52 Duroc HIGH (increased backfat thickness, intramuscular fat and saturated and monounsaturated fatty acids contents) and LOW pigs (opposite phenotype, with an increased polyunsaturated fatty acids content).

**Results:**

Our analysis revealed that 10.9% of genes expressed in the GM muscle generate alternative mRNA isoforms, with an average of 2.9 transcripts per gene. By using two different pipelines, one based on the CLC Genomics Workbench and another one on the STAR, RSEM and DESeq2 softwares, we have identified 10 mRNA isoforms that both pipelines categorize as differentially expressed in HIGH vs LOW pigs (*P*-value < 0.01 and ±0.6 log_2_fold-change). Only five mRNA isoforms, produced by the *ITGA5, SEMA4D, LITAF, TIMP1* and *ANXA2* genes, remain significant after correction for multiple testing (*q-*value < 0.05 and ±0.6 log_2_fold-change), being upregulated in HIGH pigs.

**Conclusions:**

The increased levels of specific *ITGA5*, *LITAF*, *TIMP1* and *ANXA2* mRNA isoforms in HIGH pigs is consistent with reports indicating that the overexpression of these four genes is associated with obesity and metabolic disorders in humans. A broader knowledge about the functional attributes of these mRNA variants would be fundamental to elucidate the consequences of transcript diversity on the determinism of porcine phenotypes of economic interest.

**Electronic supplementary material:**

The online version of this article (10.1186/s12864-018-4515-2) contains supplementary material, which is available to authorized users.

## Background

Recent estimates indicate that in mammals, at least 70% of genes have multiple polyadenylation sites, > 50% of genes have alternative transcription start sites and nearly 95% of genes undergo alternative splicing (AS) yielding multiple messenger ribonucleic acid (mRNA) isoforms [1, 2]. The use of alternative transcriptional initiation and/or termination sites can produce diverse pre-mRNAs, which can further be subjected to AS yielding a broad array of mRNA isoforms that are derived from a single gene. A recent study indicated that alternative transcription start and termination sites, rather than AS, encompasses most of tissue-dependent exon usage [1]. Transcripts produced by any of the mechanisms mentioned above might contribute to differences between tissues or cells by modifying protein structure and expression [3–5]. Indeed, the differential expression of mRNA isoforms has been associated with a broad array of physiological and pathological conditions in humans [3, 4] and domestic species [5, 6].

The important consequences of transcript diversity on porcine phenotypes of economic interest have been recently evidenced in a couple of studies. In White Duroc × Erhualian F_2_ intercross pigs, a mutation in a splice acceptor site of intron 9 (g.8283C > A) of the porcine phosphorylase kinase catalytic subunit gamma 1 (*PHKG1)* gene has been shown to drive the synthesis of an aberrant transcript subjected to nonsense-mediated decay [7]. This results in the inactivation of this enzyme, which plays a key role in the degradation of glycogen, and in the production of a low quality meat with a poor water-holding capacity [7]. Moreover, Koltes et al. [8], identified a mutation located in the pig guanylate binding protein 5 (*GBP5)* gene that introduces a new splice acceptor site that results in the insertion of five additional nucleotides, thus altering the open reading frame and introducing a premature stop-codon. This mutation has a major effect on the host response to the porcine respiratory and reproductive syndrome virus [8].

Transcript diversity of the porcine muscle has been poorly characterized so far and the majority of studies comparing the transcriptomes of pigs with distinct phenotypic attributes have just focused on global differences in gene expression, rather than identifying the specific transcripts that are differentially expressed (DE) [9–12]. The goals of the current experiment were to provide a first picture of transcript diversity in the *gluteus medius* (GM) muscle of pigs as well as to identify mRNA isoforms that are DE in the GM muscle of Duroc swine with distinct growth and fatness profiles.

## Methods

### Animal material

The muscle transcriptomes of 56 Duroc pigs, retrieved from a population of 350 individuals distributed in 5 half-sib families, were analyzed using RNA-sequencing (RNA-seq) (Additional file 1: Table S1). As previously reported by Gallardo et al. [13], barrows were transferred to the IRTA-CCP experimental test station after weaning (3–4 weeks of age) and bred under normal intensive conditions. In the first stage of fattening (up to 90 kg of live weight, around 150 days of age) barrows were fed ad libitum a standard diet with 18% protein, 3.8% fiber, 7.0% fat, 1.0% lysine, and 0.3% methionine (net energy concentration: 2450 kcal/kg). In the last period of fattening (i.e. 30–40 days before slaughter) animals were fed ad libitum a standard diet with 15.9% protein, 4.5% fiber, 5.2% fat, 0.7% lysine, and 0.2% methionine (net energy concentration: 2375 kcal/kg). Pigs were slaughtered when they reached ≈ 122 kg live weight (i.e. at an age of 180–200 days approximately). Backfat and ham fat thickness were measured with a ruler in the cutting room 24 h after slaughtering. Lean meat content was estimated on the basis of fat and muscle thickness data measured with an Autofom ultrasound device. Samples of the GM muscle were retrieved, snap frozen in liquid nitrogen and stored at − 80 °C. A near infrared transmittance device (NIT, Infratec 1625, Tecator Hoganas, Sweden) was employed to determine intramuscular fat content. The determination of fatty acid composition was achieved with a technique based on the gas chromatography of methyl esters [14]. As reported by Gallardo and coworkers [13], blood samples were obtained at 190 days and a variety of enzymatic methods were used to determine cholesterol (cholesterol oxidase-based method), high-density lipoprotein (immunoinhibition method) and triglyceride concentrations (glycerol kinase reaction). Low density lipoprotein concentration was calculated according to the equation of Friedewald et al. [15].

Principal component analysis based on the 13 traits listed in Table 1 was performed in order to select pigs with distinct growth and fatness phenotypes (HIGH and LOW pigs) [10]. When compared with LOW pigs, the HIGH (*n* = 28) ones showed a higher live weight, backfat thickness and intramuscular fat content and also displayed increased serum lipid concentrations and muscle saturated (SFA) and monounsaturated (MUFA) fatty acids contents (Table 1). On the other hand, LOW pigs (*n* = 28), were lighter, leaner and had a higher muscle polyunsaturated fatty acids (PUFA) content than HIGH pigs.

**Table 1:** Mean values ± standard deviation (SD) for 13 phenotypes recorded in HIGH and LOW Duroc pigs

Phenotypes	HIGH group (*N* = 28)	LOW group (*N* = 28)
	Mean ± SD	Mean ± SD
Carcass traits		
LW - Live weight (kg)	130.90 ± 9.46 ^a^	110.75 ± 16.62 ^b^
BFTiv - Backfat thickness in vivo (mm)	28.74 ± 3.47 ^a^	18.76 ± 3.90 ^b^
BFT - Backfat thickness 3rd-4th ribs (mm)	47.07 ± 11.94 ^a^	33.89 ± 10.03 ^b^
HFT - Ham fat thickness (mm)	28.02 ± 2.70 ^a^	20.97 ± 3.56 ^b^
LEAN - Lean content (%)	39.17 ± 5.15 ^a^	45.48 ± 4.21 ^b^
Meat quality traits (*gluteus medius*)		
IMF - Intramuscular fat content (%)	7.27 ± 1.70 ^a^	3.69 ± 0.93 ^b^
SFA - Saturated fatty acids content (%)	38.70 ± 1.41 ^a^	34.76 ± 1.30 ^b^
PUFA - Polyunsaturated fatty acids content (%)	14.71 ± 3.08 ^a^	27.82 ± 4.40 ^b^
MUFA - Monounsaturated fatty acids content (%)	46.58 ± 2.67 ^a^	37.4 ± 4.30 ^b^
Serum lipid levels - 190 days		
CHOL - Total cholesterol (mg/dL)	161.11 ± 30.32 ^a^	104.17 ± 16.40 ^b^
HDL - HDL-cholesterol (mg/dL)	61.12 ± 8.58 ^a^	42.92 ± 9.19 ^b^
LDL - LDL-cholesterol (mg/dL)	86.34 ± 29.32 ^a^	50.57 ± 15.12 ^b^
TG - Triacylglycerides (mg/dL)	68.07 ± 26.28 ^a^	50.71 ± 29.70 ^b^

Means with different letters are significantly different (*P*-value < 0.05), t-test for: LW, IMF, MUFA, CHOL and LDL; Wilcoxon test for: BFTiv, BFT, LEAN, SFA, PUFA, HDL and TG

### RNA isolation, library construction and sequencing

Each muscle sample (*N* = 56, 28 HIGH and 28 LOW) was individually submerged in liquid nitrogen and grinded with a mortar and a pestle to produce a homogenous powder. This powder was submerged in TRIzol reagent (Thermo Fisher Scientific, Barcelona, Spain) and homogenized with a Polytron device (IKA, Staufen, Germany). Total RNA was purified with the Ambion RiboPure kit (Thermo Fisher Scientific, Barcelona, Spain) by following the instructions of the manufacturer. RNA samples were resuspended in a buffer solution provided in the kit and kept at − 80 °C until use. RNA quantification and purity were assessed with a Nanodrop ND-1000 spectrophotometer (Thermo Fisher Scientific, Barcelona, Spain), while integrity was checked with a Bioanalyzer-2100 equipment (Agilent Technologies, Santa Clara, CA). All samples showed an RNA integrity number above 7.5. Sequencing libraries were prepared with the TruSeq RNA Sample Preparation Kit (Illumina, San Diego, CA) and sequenced in a paired-end mode (2 × 75 bp), multiplexing two samples in each sequencing lane, on a HiSeq2000 Sequencing System (Illumina, San Diego, CA). Library preparation and sequencing were developed according to the protocols recommended by the manufacturer.

### Differential expression analyses of mRNA isoforms between HIGH and LOW pigs

Adaptors and low quality bases were trimmed from sequences by using Trimmomatic [16] with default parameters. Quality control of sequences in FASTQ and BAM format was assessed with the FASTQC software (Babraham Bioinfomatics, http://www.bioinformatics.babraham.ac.uk/projects/fastqc/). Sequence quality was measured by taking into account sequence-read lengths and base-coverage (distribution = 75 bp, 100% coverage in all bases), nucleotide contributions and base ambiguities (GC-content ~ 50%, ~ 25% of A, T, G and C nucleotide contributions and an ambiguous base-content < 0.1%) and a Phred score higher than 30 (i.e. base-calling accuracy larger than 99.9%). All samples, except four, passed the quality control parameters, so our final data set consisted of 52 animals. With the aim of minimizing the rate of false positives, we used two different pipelines in the analysis of differential expression. In the first pipeline, read mapping and counting were carried out with CLC Genomics Workbench 8.5 (CLC Bio, Aarhus, Denmark, https://www.qiagenbioinformatics.com/). In the second pipeline, reads were mapped with Spliced Transcripts Alignment to a Reference (STAR) v. 2.4 [17], counted with the RNA-Seq by Expectation Maximization (RSEM) software v. 1.3 [18] and differential expression was analysed with DESeq2 [19]. We considered as DE mRNA isoforms those simultaneously identified with the two pipelines.

#### Pipeline 1 (CLC genomics workbench)

The Large Gap Mapper (LGM) tool of CLC Genomics Workbench 8.5 was used to map the reads. This tool can map sequence reads that span introns without requiring prior transcript annotations. In this way, the LGM tool finds the best match for a given read. If there is an unaligned end which is long enough for the mapper to handle (17 bp for standard mapping) this segment of the read is re-mapped with the standard read mapper of the CLC Genomics Workbench. This process is repeated until no reads have unaligned ends that are longer than 17/18 bp. In our study, short sequence reads were mapped and annotated by using as template the pig reference genome version 10.2 (*Sscrofa* 10.2 - http://www.ensembl.org/info/data/ftp/index.html). Additional details can be found in http://resources.qiagenbioinformatics.com/manuals/transcriptdiscovery/208/index.php?manual=Large_gap_mapper.html. For mapping purposes, we considered alignments with a length fraction of 0.7 and a similarity fraction of 0.8. Two mismatches and three insertions and deletions per read were allowed. The quantification of mRNA isoform levels by the CLC Genomics Workbench follows a count-based model, where reads are counted on small counting units (exons), instead of the whole transcript unit, and the two possible splicing outcomes (inclusion and/or exclusion) are tested for each counting unit. Normalized count values are transformed on a decimal logarithmic scale. Statistical analysis of differential expression of splicing variants is based on an empirical analysis of digital gene expression [20], that implements an ‘Exact Test’ for two-group comparisons, assuming a negative binomial distribution and an overdispersion caused by biological variability estimated at 5%.

#### Pipeline 2 (STAR/RSEM/DESeq2)

The STAR software v. 2.4 [17] was employed to map the reads generated in the RNA-Seq experiment. The STAR algorithm comprises two main steps. First, a sequential maximum mappable seed search is carried out. For instance, if a read contains a single splice junction, a first seed is mapped to a donor splice site and the unmapped portion of the read is mapped again (in this case to an acceptor splice site). Subsequently, STAR builds alignments of the entire read sequence by stitching together all the seeds that were aligned to the genome in the first step [17]. In our study, the parameters employed in STAR mapping were those reported by Zhang et al. [21] and the pig reference genome v. 10.2 (*Sscrofa* 10.2) was used as template.

Once reads were mapped, they were counted with the RSEM v. 1.3 [18] software by using default parameters with the option “--paired-end” and considering the porcine gene annotation file and the pig *Sscrofa* 10.2 genome sequence. RSEM generates a set of reference transcript sequences and subsequently a set of RNA-Seq reads are aligned to these reference transcripts [18]. Alignments generated with this procedure are used to infer transcript abundances by computing maximum likelihood abundance estimates with the Expectation-Maximization algorithm [18]. Credibility intervals at 95% are built with a Bayesian approach implemented in RSEM. Additional details can be found in Li et al. [18]. Read counts associated with each specific mRNA isoform were employed to carry out analysis of differential expression with DESeq2 [19]. DESeq2 assumes that read counts follow a negative binomial distribution, for each gene *i* and for each sample *j*, with a mean μ_ij_ and a dispersion value α_i_. Means are proportional to the amounts of complementary deoxyribonucleic acid (cDNA) fragments corresponding to each gene scaled by a normalization factor. Gene-wise dispersion values are calculated with a maximum likelihood approach and subsequently they are shrunk towards a set of predicted dispersion values with an empirical Bayes approach. Subsequently, DESeq2 shrinks log_2_ fold-change (FC) estimates, with an empirical Bayes procedure [19], to reduce variance due to noisiness issues of genes that are poorly expressed. Finally, a Wald test is used to infer if shrunk log_2_FC estimates (and their standard errors) are significantly different from zero. In the Wald test, the shrunken estimate of the log_2_FC is divided by its standard error, generating a *z*-statistic that can be compared to a standard normal distribution [19].

### Transcript annotation

To classify splicing events with the SUPPA [22] and Splicing Express [23] softwares, genome BAM files were generated with the STAR software [17], by using the same parameters described above. These BAM files were employed to assemble transcripts with Cufflinks [24], taking as a reference the *Sscrofa* 10.2 genome, and a master transcriptome was generated with Cuffmerge. The SUPPA software annotates AS events from a general input annotation file generated with Cuffmerge. The AS event types considered by SUPPA are: exon skipping, alternative 5′ and 3′ splice sites, and intron retention. For each event, SUPPA calculates the inclusion parameter Ψ, which is defined as the ratio of the abundance of transcripts that include one form of the event over the abundance of the transcripts that contain either form of the event. On the other hand, the Splicing Express software uses a well-annotated set of reference sequences to detect different AS events from a transcriptome data (GTF file) input file i.e. exon skipping, intron retention and alternative 5′ and 3′ splicing borders. Splicing Express clusters expressed transcripts to identify their gene of origin and identifies AS events by using an algorithm based on the pairwise comparison. Besides, expressed sequences are represented as binary sequences (exons = 1, introns = 0) that are pairwisely compared thus generating numerical patterns which reflect their splicing differences. Finally, a graphic representation of the expression level is created for each gene and for each identified AS event [23].

Transcript type annotation of porcine GM mRNA isoforms was retrieved from the BioMart database, available in the Ensembl database (http://www.ensembl.org/biomart/martview/). Gene Ontology (GO) Enrichment Analysis was performed by using the Panther database v. 12.0 (http://www.pantherdb.org/) with the data set of 87 genes simultaneously detected by both pipelines (*P*-value < 0.05) as producing mRNA isoforms DE in HIGH vs LOW pigs.

### Validation of differentially expressed mRNA isoforms by RT-qPCR

Differential expression of mRNA isoforms was validated for the MAF BZIP transcription factor F (*MAFF*), stearoyl-CoA desaturase (*SCD*), retinoic acid receptor γ (*RXRG*) and integrin α5 (*ITGA5*) genes by reverse transcription-quantitative polymerase chain reaction (RT-qPCR). Primers spanning exon-exon boundaries, or alternatively binding at different exons (in order to avoid the amplification of residual contaminating genomic DNA), and complementary to exonic regions that define specific isoforms were designed with the Primer Express software (Applied Biosystems) (Additional file 2: Table S2). One μg of total RNA from 14 pigs (7 from each group - HIGH and LOW), selected at random from the global population of 52 pigs, was used as template for cDNA synthesis. The reverse transcription reaction was carried out with the High-Capacity cDNA Reverse Transcription Kit (Applied Biosystems, Foster City, CA) in a final volume of 20 μl. Quantitative PCR reactions included 7.5 μl of SYBR Select Master Mix, 300 nM of each primer and 3.75 μl of a 1:25 dilution of the cDNA in a final volume reaction of 15 μl. Three genes e.g. β-actin (*ACTB*), TATA-Box binding protein (*TBP*) and hypoxanthine phosphoribosyltransferase 1 (*HPRT1*) were used as endogenous controls. The PCR thermal cycle involved one denaturing step at 95 °C for 10 min plus 40 cycles of 15 s at 95 °C and 1 min at 60 °C. Reactions were run in a QuantStudio 12 K Flex Real-Time PCR System (Applied Biosystems, Foster City, CA). A melting curve analysis i.e. 95 °C for 15 s, 60 °C for 15 s and a gradual increase in temperature, with a ramp rate of 1% up to 95 °C, followed by a final step of 95 °C for 15 s, was performed after the thermal cycling protocol to ensure the specificity of the amplification. We made sure that housekeeping and target genes had comparable amplification efficiencies (90–110%) by performing standard curve assays with serial 1:5 dilutions. Gene expression levels were quantified relative to the expression of endogenous controls by employing an optimized comparative Ct (2^-ΔΔCt^ method) value approach [25] implemented in the Thermo Fisher Cloud (Thermo Fisher Scientific, Barcelona, Spain). Each sample was analysed in triplicate. All results were evaluated using RT-qPCR data analysis software (Thermo Fisher Cloud, Thermo Fisher Scientific, Barcelona, Spain). The sample displaying the lowest expression was used as calibrator. Differential expression was assessed with a Student’s t-test.

## Results

After quality control analysis, we used a final dataset of 52 GM muscle samples equally distributed between the HIGH (*N* = 26) and LOW (*N* = 26) groups. RNA-sequencing of these samples generated an average of 66 million paired-end reads per sample. The majority of reads (72.8%, CLC Bio; 89% STAR software) were successfully mapped to the pig *Sscrofa* 10.2 genome assembly. The mean mapping proportions obtained with CLC Bio were 91.4% and 8.6% for reads corresponding to exonic and intronic regions, respectively. When using STAR, 79.3% of the mapped reads were located in exons and 6.5% in introns. The remaining (14.2%) reads mapped to intergenic regions.

In the CLC Bio analysis, we found evidence of the existence of alternative transcripts in 2066 genes (11.7% of protein-coding genes expressed in the GM muscle of HIGH and LOW swine) which produced 5835 mRNA isoforms (2.8 transcripts per gene). In contrast, the STAR software detected 1430 genes (10.2% of expressed protein-coding genes) yielding 4391 different transcripts (3.0 transcripts per gene). Only 5.0% of alternative transcript variants were potentially subject to nonsense-mediated decay (Additional file 3: Table S3). Interestingly, 93% of the genes identified by STAR/RSEM/DESeq2 as displaying alternative transcripts were also detected with CLC Bio. Analysis of transcriptomic data with the SUPPA software [22] evidenced that exon skipping is the most prevalent AS event, while intron retention is the rarest one, i.e. they comprise 36.7% and 12.2% of all GM AS events, respectively (Additional file 4: Table S4). Similar results were obtained with the Splicing Express software [23] (Additional file 4: Table S4), i.e. exon skipping was the most prevalent AS event (41.1%) and intron retention the least favoured one (12.7%).

We used two different pipelines (CLC Bio and STAR/RSEM/DESeq2) to detect DE mRNA isoforms in HIGH vs LOW pigs. Combination of such data sets made possible to identify 104 alternative transcripts and 87 genes that were simultaneously detected by both pipelines (*P*-value < 0.05) (Additional file 5: Table S5). A more stringent analysis (*P*-value < 0.01 and ±0.6 log_2_FC) ascertained 10 DE transcripts (corresponding to 10 genes) concurrently discovered by both pipelines (Table 2). Five of these transcripts remained significant after correction for multiple testing (*q-*value < 0.05 and ±0.6 log_2_FC; Table 2). In general, differential expression only affected one isoform and, more particularly, that showing a predominant pattern of expression (Tables 2, 3 and Additional file 6: Table S6). In order to validate the accuracy of our RNA-Seq approach, we measured the expression of four DE mRNA isoforms (*ITGA5, SCD, RXRG* and *MAFF*) by RT-qPCR analysis (Additional file 7: Figure S1). A significant differential expression was confirmed for two splicing variants e.g. *ITGA5* (4445 bp) and *SCD* (5585 bp) genes (*P*-value < 0.04). Besides, a strong statistical tendency was observed for the *RXRG* (544 bp) gene (*P*-value = 0.06). In contrast, the *MAFF* (2145 bp*, P*-value = 0.23) did not show a statistically significant differential expression, though RT-qPCR data reflected the same trends (FC and raw abundance estimates) detected by RNA-seq.

**Table 2:** Splicing variants that are differentially expressed (*P-*value < 0.01 and ±0.6 log_2_Fold-Change) in the *gluteus medius* muscle of HIGH (*N* = 26) vs LOW pigs (N = 26)^a^

Feature ID	Feature transcript ID	Transcript ID	Length (bp)	Relative expression mean (%)	CLC Bio			STAR/RSEM/DESeq2		
					Log_2_(FC)	*P*-value	*q-*value	Log_2_(FC)	*P*-value	*q-*value^b^
**ENSSSCG00000000293**	**ENSSSCT00000000314**	***ITGA5–201***	**4445**	**99.00**	**0.85**	**2.64E-05**	**2.56E-02**	**0.85**	**2.54E-06**	**1.21E-03**
ENSSSCG00000001252	ENSSSCT00000001367	*UBD-201*	1281	75.81	−0.72	1.37E-03	2.29E-01	−0.83	7.73E-04	NA
ENSSSCG00000003079	ENSSSCT00000003416	*PVR-202*	1167	48.41	0.77	7.76E-03	5.94E-01	0.66	9.27E-03	1.27E-01
**ENSSSCG00000004578**	**ENSSSCT00000005057**	***ANXA2–202***	**1455**	**99.36**	**0.70**	**3.32E-06**	**6.56E-03**	**0.69**	**4.56E-06**	**1.58E-03**
**ENSSSCG00000012277**	**ENSSSCT00000013426**	***TIMP1–001***	**931**	**97.85**	**2.57**	**4.61E-07**	**1.68E-03**	**0.79**	**2.01E-04**	**1.32E-02**
ENSSSCG00000012653	ENSSSCT00000013834	*ZDHHC9–209*	2967	17.79	0.77	3.29E-03	3.75E-01	0.77	2.56E-03	6.11E-02
ENSSSCG00000013579	ENSSSCT00000014831	*CD209–001*	1042	95.10	1.04	7.84E-05	4.18E-02	1.01	2.09E-05	NA
ENSSSCG00000006328	ENSSSCT00000033501	*RXRG-202*	544	17.68	−0.70	4.09E-04	1.18E-01	−0.76	9.31E-05	8.98E-03
**ENSSSCG00000009584**	**ENSSSCT00000034286**	***SEMA4D-208***	**487**	**8.57**	**1.40**	**2.75E-06**	**6.53E-03**	**1.09**	**5.87E-05**	**6.59E-03**
**ENSSSCG00000024982**	**ENSSSCT00000036552**	***LITAF-201***	**2190**	**88.44**	**1.01**	**1.02E-04**	**4.38E-02**	**0.79**	**1.21E-04**	**1.04E-02**

^a^Differentially expressed mRNA isoforms that remained significant after correction for multiple testing (*q-*value < 0.05 and ±0.6 log_2_Fold-Change) are shown in bold. A positive log_2_FC means that the gene is upregulated in HIGH pigs

^b^For multiple testing correction, DESeq2 carries out a filtering step based on the average expression strength of each gene across all samples with the aim of discarding genes which are likely to loose significance after correcting for multiple testing. The purpose of this filtering step is to increase statistical power by reducing the list of candidate genes to be tested. The *q-*values of the genes which do not pass the filtering step are set to NA

**Table 3:** Relative expression of the set of isoforms of four loci (*TIMP1, ITGA5, ANXA2* and *LITAF*) in HIGH (N = 26) vs LOW (N = 26) pigs^a^

Feature ID	Feature transcript ID	Transcript ID	Length (bp)	Type^b^	Relative expression mean (%)	CLC Bio			STAR/RSEM/DESeq2		
						Log_2_ (FC)	*P-*value	*q-*value	Log_2_ (FC)	*P-*value	*q-*value^c^
ENSSSCG00000004578	ENSSSCT00000034155	*ANXA2–201*	1609	Protein coding	0.64	0.21	6.77E-01	1.00E + 00	0.07	9.20E-01	NA
**ENSSSCG00000004578**	**ENSSSCT00000005057**	***ANXA2–202***	**1455**	**Protein coding**	**99.36**	**0.70**	**3.32E-06**	**7.23E-03**	**0.69**	**4.56E-06**	**1.58E-03**
**ENSSSCG00000000293**	**ENSSSCT00000000314**	***ITGA5–201***	**4445**	**Protein coding**	**99.00**	**0.85**	**2.64E-05**	**2.82E-02**	**0.85**	**2.54E-06**	**1.21E-03**
ENSSSCG00000000293	ENSSSCT00000035821	*ITGA5–203*	1255	NMD	0.34	−0.03	9.18E-01	1.00E + 00	–	–	–
ENSSSCG00000000293	ENSSSCT00000034427	*ITGA5–204*	1013	NMD	0.71	−0.08	1.00E + 00	1.00E + 00	–	–	–
ENSSSCG00000000293	ENSSSCT00000033141	*ITGA5–205*	766	NMD	0.48	−0.29	1.00E + 00	1.00E + 00	−0.01	9.97E-01	NA
**ENSSSCG00000012277**	**ENSSSCT00000013426**	***TIMP1–001***	**931**	**Protein coding**	**97.86**	**2.57**	**4.61E-07**	**1.85E-03**	**0.79**	**2.01E-04**	**1.32E-02**
ENSSSCG00000012277	ENSSSCT00000033796	*TIMP1–002*	598	Protein coding	0.38	1.12	5.56E-01	1.00E + 00	0.38	8.98E-01	NA
ENSSSCG00000012277	ENSSSCT00000034602	*TIMP1–003*	641	Protein coding	1.57	1.19	8.47E-01	1.00E + 00	−0.19	7.39E − 01	NA
ENSSSCG00000012277	ENSSSCT00000036308	*TIMP1–004*	173	Protein coding	0.19	3.05	4.21E-01	1.00E + 00	0.18	9.52E-01	NA
**ENSSSCG00000024982**	**ENSSSCT00000036552**	***LITAF-201***	**2190**	**Protein coding**	**88.45**	**1.01**	**1.02E-04**	**4.82E-02**	**0.79**	**1.21E-04**	**1.04E-02**
ENSSSCG00000024982	ENSSSCT00000025103	*LITAF-202*	2370	Protein coding	11.55	-0.04	1.00E + 00	1.00E + 00	0.49	7.52E-01	NA

^a^Differentially expressed mRNA isoforms that remained significant after correction for multiple testing (*q-*value < 0.05 and ±0.6 log_2_Fold-Change) are shown in bold. A positive log_2_FC means that the gene is upregulated in HIGH pigs

^**b**^*NMD* Nonsense-mediated mRNA decay

^c^For multiple testing correction, DESeq2 carries out a filtering step based on the average expression strength of each gene across all samples with the aim of discarding genes which are likely to loose significance after correcting for multiple testing. The purpose of this filtering step is to increase statistical power by reducing the list of candidate genes to be tested. The *q-*values of the genes which do not pass the filtering step are set to NA

**Fig. 1:**
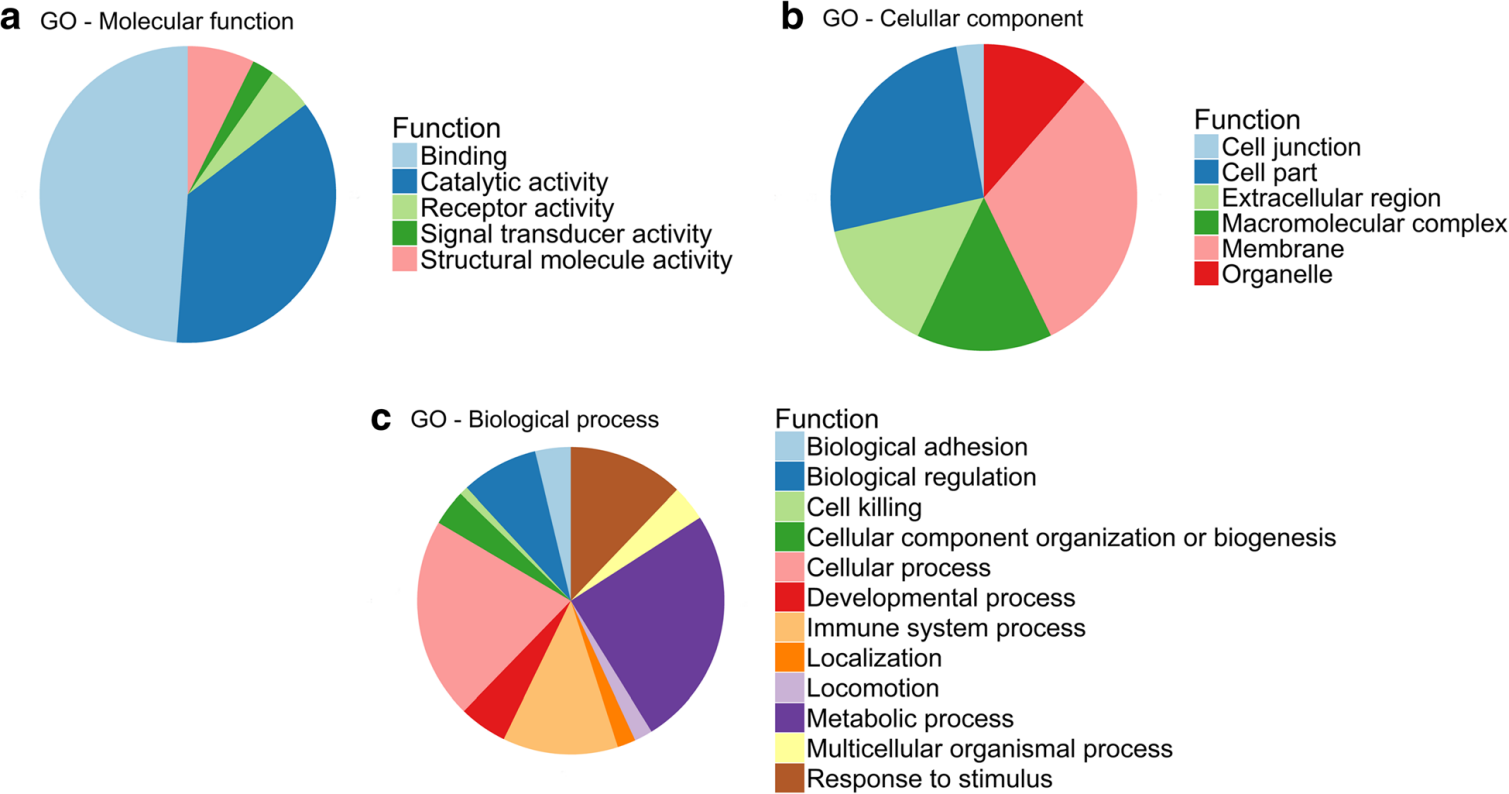
Functional classification of genes with differentially expressed (*P*-value < 0.05) mRNA isoforms identified with the CLC Genomics Workbench and STAR/RSEM/DESeq2 pipelines in the *gluteus medius* muscle of HIGH vs LOW pigs. **a** molecular function, **b** cellular components and **c** biological processes. Categorizations were based on information provided by the online resource PANTHER classification system (http://www.pantherdb.org)

We carried out a GO analysis of the data set of 87 genes producing alternative transcripts (*P-*value < 0.05). We did not analyse the two other data sets (10 genes, *P*-value < 0.01 and ±0.6 log_2_FC; 5 genes, *q-*value < 0.05 and ±0.6 log_2_FC) because they are too small. The main molecular functions identified in the data set of 87 genes were *Binding* and *Catalytic activity* (Fig. 1a). These results are consistent with those of Lindholm et al. [26], who found that the main functions of mRNA encoding genes expressed in the human skeletal muscle are also related with binding and catalytic activity. The top GO terms of the cellular component GO category were *Membrane* and *Cell part* (Fig. 1b), while *Metabolism* and *Cellular process* were the most common biological processes amongst genes producing alternative transcripts (Fig. 1c). These results agree well with previous data obtained in humans, mouse and cow [23]. These functional processes are remarkably unspecific, thus probably reflecting the heterogeneous biological roles of genes expressing alternative transcripts.

## Discussion

About 10.9% (average of CLC Bio and STAR results) of pig genes expressed in the GM muscle produced alternative transcripts, as opposed to 95% of genes detected in a broad array of human tissues [2]. Besides, the average number of mRNA isoforms per gene in the porcine skeletal muscle was 2.9 (average of CLC Bio and STAR results). The analysis of transcript diversity in the human skeletal muscle revealed a similar pattern, with an average of 2 isoforms per gene [26], a figure that is clearly below the average transcript diversity (5.4 isoforms/gene) found in other human tissues [27]. Such feature might be due to the fact that in the skeletal muscle there is a reduced number of transcripts (e.g. myofibrillar proteins) that encompass a disproportionate fraction of the total transcriptome [26]. Besides that, Taneri et al. [28] predicted a lower quantity of transcripts per gene in primary tissues e.g. skeletal muscle. In cattle, Chacko et al. [29] observed that 21% of genes are alternatively spliced, and similar percentages were observed by Kim et al. [30] in cows (26%) and dogs (14%).

The types of splicing events detected in the porcine muscle have been compiled in Additional file 4: Table S4. Exon skipping was the most frequent AS event predicted by SUPPA and Splicing Express softwares, followed by the use of alternative 5′ splice (SUPPA) and 3′ splice (Splicing Express) sites. Deep sequencing of 15 human tissues and cell lines was consistent with these findings, thus demonstrating that exon skipping was the most frequent AS event, followed by the use of alternative 3′ splice and 5′ splice sites [31]. In accordance with results obtained in humans [31], the less frequent event was intron retention (Additional file 4: Table S4). Besides, only 5.0% of produced transcripts were predicted to undergo nonsense-mediated decay. In humans, nonsense-mediated decay and nuclear sequestration and turnover of intron-retention transcripts have been involved in the downregulation of genes in tissues where they do not have a relevant physiological role [32]. Though SUPPA and Splicing Express yielded consistent results about the relative importance of distinct AS event categories in the porcine skeletal muscle, the sets of genes identified by these two softwares as yielding alternative transcripts were quite different. The proportions of genes overlapping the SUPPA and Splicing Express data sets classified according to the type of AS event were: exon skipping = 27%, alternative 5′ =24%, 3′ splice sites = 20% and intron retention = 14%. Though we do not have a straightforward explanation for these discrepancies, we hypothesize that they might be due to the existence of relevant differences in the assumptions and algorithms on which these two softwares are based.

Estimating isoform mRNA abundance is a challenging task and results may vary depending on the bioinformatics approach employed in differential expression analysis [33]. One of the main factors influencing the outcome of differential mRNA isoform expression is the quality and completeness of the transcript assembly [33]. The mRNA isoform annotation of the pig genome is still incomplete and obviously this might affect the results of our analysis, but we have not attempted to reconstruct transcripts because bioinformatic and statistical approaches to do so are not very robust and they may lead to inaccurate transcript quantitations [34].

In order to obtain results as much precise as possible, we have used two different pipelines to identify DE mRNA isoforms and we have considered as genuine differential expression events those identified by both approaches. This combined analysis highlighted the existence of five genes with DE mRNA isoforms that remained significant after correction for multiple testing (*q-*value < 0.05, ± 0.6 log_2_FC). It is worth to highlight that the DE isoform (487 bp) of the pig semaphorin 4D (*SEMA4D*) gene is annotated, in the Ensembl database (*Sscrofa* 10.2 assembly; https://www.ensembl.org), as truncated in its 3’end. In the human *SEMA4D* gene, there are 13 protein-encoding mRNA isoforms and five of them are also truncated in their 3’ends (*GRCh38.p10* assembly; https://www.ensembl.org). The existence of truncated transcripts is due to the inability of conventional RNA-seq experiments to define the ends of genes with high precision [35]. Moreover, automated gene prediction is a difficult task and, in consequence, first-pass annotations can be quite inaccurate [35]. Obviously, the analysis of the differential expression of mRNA isoforms strongly depends on the accuracy of transcript annotation, so the results presented in the current work need to be interpreted with this caveat in mind.

By examining Tables 2 and 3, we have noticed that the differential expression of mRNA isoforms might have different functional consequences depending on the gene under consideration. For instance, in the case of the *ITGA5* and TIMP metallopeptidase inhibitor 1 (*TIMP1*) genes the DE mRNA isoform encodes a protein that is longer than the proteins encoded by the remaining mRNA isoforms recorded in the Ensembl database (*Sscrofa* 10.2 assembly; https://www.ensembl.org). With regard to *ITGA5*, the DE isoform (4445 bp) encodes a full length protein of 1057 amino acids (aa) and it has a predominant pattern of expression (99%), while the remaining porcine *ITGA5* isoforms reported in the Ensembl database correspond to processed transcripts or transcripts subject to nonsense mediated decay. Similarly, in humans there is one major *ITGA5* isoform (4444 bp), another one that might encode a protein but it is truncated in its 5’end, and eleven isoforms that correspond to processed transcripts, retained introns and transcripts subject to nonsense mediated decay (https://www.ensembl.org). Concerning the *TIMP1* gene, the DE isoform (931 bp) encodes a full length protein of 207 aa that is longer than the proteins encoded by other isoforms (*Sscrofa* 10.2 assembly; https://www.ensembl.org): 195 aa (but incomplete 5’end), 123 aa and 38 aa (but incomplete 3’end). If we compare the 207 aa (931 bp transcript) and the 123 aa (598 bp transcript) *TIMP1* isoforms, the latter lacks a central part of the protein (from aa site 68 to 151), a feature that involves the loss of four of the six disulfide bridges which stabilize the fold of the molecule and of two aa residues (sites 68 and 69) which bind to the catalytic zinc [36, 37]. These observations imply that the two 207 aa and 123 aa porcine protein isoforms are expected to be very different at the functional level.

A different case is represented by the annexin A2 (*ANXA2*) and lipopolysaccharide-induced TNF-alpha factor (*LITAF*) genes in which the DE mRNA isoform (*ANXA2*: 1455 bp, *LITAF*: 2190 bp) is shorter than the longest annotated transcript (*ANXA2*: 1609 bp, *LITAF*: 2370 bp, Table 3) but both encode proteins of identical length (*ANXA2*: 339 aa, *LITAF*: 161 aa, *Sscrofa* 10.2 assembly; https://www.ensembl.org). This situation is comparable to what has been reported in humans for the *ANXA2* (1676 bp, 1444 bp and 1435 bp isoforms encoding a protein of 339 aa) and *LITAF* (six different isoforms e.g. 2632 bp, 2467 bp, 2356 bp, 1118 bp, 717 bp and 603 bp mRNAs encoding a protein of 161 aa) genes (*GRCh38.p10* assembly; http://www.ensembl.org). Though proteins with an identical length and sequence composition should be functionally equivalent, differences in transcript length might affect mRNA translatability (e.g. presence of short upstream open reading frames in the 5’UTR), stability (e.g. formation of stable stem-loops, presence of microRNA binding sites and of AU-rich elements) and cell localization [38].

The upregulation of certain mRNA isoforms of the *ITGA5*, *TIMP1*, *ANXA2* and *LITAF* genes in HIGH pigs is relevant because these four genes have been implicated in human obesity and diabetes. For instance, high glucose concentrations induce the overexpression of the fibronectin receptor, an heterodimer whose α-chain is encoded by the *ITGA5* gene [39]. Moreover, *TIMP1* expression is increased in the serum and adipose tissue of obese mouse models [40]. There is also evidence that the knockout of the *ANXA2* gene in mice involves an hypotrophy of the white adipose tissue due to reduced fatty acid uptake [41], and *LITAF* mRNA is overexpressed in overweight and obese humans [42]. In summary, the upregulation of these four genes in HIGH swine is consistent with the increased fatness and live weight of these pigs and suggests that the differential expression of specific mRNA isoforms might contribute to the phenotypic differences observed in HIGH vs LOW pigs.

Finally, we would like to discuss a third case in which DE mRNA isoforms encode proteins that are shorter than the canonical full-length protein. We have observed that a 3155 bp transcript corresponding to the porcine ubiquitin specific peptidase 2 (*USP2*) gene and encoding a 396 aa protein is upregulated in HIGH pigs (Additional file 5: Table S5). In the Ensembl database (http://www.ensembl.org, *Sscrofa* 10.2), a second mRNA isoform that encodes a 606 aa protein has been annotated. In humans, two USP2 protein isoforms of 605 aa (USP2–69) and 396 aa (USP2–45) have been reported and that there are evidences that both are able to prevent the degradation of the low density lipoprotein (LDL) receptor. The upregulation of the 396 aa *USP2* isoform in HIGH swine might constitute a mechanism to cope with the elevated serum LDL concentrations observed in this group of pigs (Table 1). It is also worth to highlight that the USP2–69 and USP2–45 isoforms might not be functionally equivalent. In *Xenopus*, for instance, USP2–45 can deubiquitylate epithelial Na^+^ channels in oocytes, while USP2–69 cannot perform such function due to differences in their N-terminal domains. In humans, functional differences have been also observed with regard to the implication of *USP2* isoforms in cell cycle progression and antiviral response [43], but unfortunately no such data are currently available for pigs.

## Conclusions

We have demonstrated that around 10.9% of genes expressed in the porcine skeletal muscle produce alternative transcripts, thus generating an average of 2.9 different mRNA isoforms per gene. Exon skipping is the most frequent splicing event, followed by the use of alternative 5′ splice sites (SUPPA) and 3′ splice sites (Splicing Express). By analysing the differential expression of mRNA isoforms in HIGH vs LOW pigs, we have demonstrated that in the GM muscle of HIGH pigs, which display an increased fatness, specific *ITGA5*, *ANXA2*, *LITAF* and *TIMP1* mRNA isoforms are upregulated. This finding is biologically meaningful because these four genes have been implicated in human obesity and metabolism [39–42]. A deeper functional characterization of these mRNA isoforms, through initiatives such as the Functional Annotation of Farm Animal Genomes project [44], will be essential to infer the consequences of their differential expression on porcine growth and fatness.

## Additional files

Additional file 1: Table S1. Distribution of the 56 animals sequenced by RNA-seq in the 5 half-sib families reported by Gallardo et al. [13]. (XLSX 9 kb)

Additional file 2: Table S2. Primers employed in the validation of four differentially expressed isoforms by RT-qPCR. (XLSX 10 kb)

Additional file 3: Table S3. Alternatively spliced mRNA isoforms identified in the porcine *gluteus medius* muscle of Duroc pigs by CLC Bio and/or STAR/RSEM/ DESEq2. (XLSX 569 kb)

Additional file 4: Table S4. Classification of alternative splicing (AS) events detected in the porcine *gluteus medius* muscle with the SUPPA and Splicing Express softwares. (XLSX 11 kb)

Additional file 5: Table S5. Differentially expressed (*P*-value < 0.05) mRNA isoforms (HIGH vs LOW pigs) found with CLC Bio and STAR/RSEM/DESeq2 softwares. (XLSX 225 kb)

Additional file 6: Table S6. Relative transcript levels of a set of isoforms corresponding to five genes expressed in the *gluteus medius* muscle of HIGH and LOW pigs identified with the CLC Bio and STAR/RSEM/DESeq2 pipelines. (XLSX 160 kb)

Additional file 7: Figure S1. Validation by RT-qPCR of the differential expression of mRNA isoforms corresponding to the *RXRG*, *SCD*, *MAFF* and *ITGA5* genes in HIGH vs LOW pigs. (PPT 132 kb)

## Abbreviations

aa: Amino acids
*ACTB*: β-actin
*ANXA2*: Annexin A2
cDNA: Complementary deoxyribonucleic acid
DE: Differentially expressed
FC: Fold-change
GBP5: Guanylate binding protein 5
GM: *Gluteus medius*
GO: Gene ontology
*HPRT1*: Hypoxanthine phosphoribosyltransferase 1
*ITGA5*: Integrin α5
LDL: Low density lipoprotein
LGM: Large Gap Mapper
*LITAF*: Lipopolysaccharide-induced TNF-α factor
*MAFF*: MAF BZIP transcription factor F
MUFA: Monounsaturated fatty acids
PUFA: Polyunsaturated fatty acids
RNA-seq: RNA-sequencing
RSEM: RNA-Seq by Expectation Maximization software
RT-qPCR: Reverse transcription-quantitative polymerase chain reaction
*RXRG*: Retinoic acid receptor γ
*SCD*: Stearoyl-CoA desaturase
*SEMA4D*: Semaphorin 4D
SFA: Saturated fatty acids
STAR: Spliced Transcripts Alignment to a Reference Software
*TBP*: TATA-Box binding protein
*TIMP1*: TIMP metallopeptidase inhibitor 1
*USP2*: Ubiquitin specific peptidase 2
