# Supplementary material for: Differential expression of mRNA isoforms in the skeletal muscle of pigs with distinct growth and fatness profiles
Source: BMC Genomics. 2018 Feb 14;19:145. doi: 10.1186/s12864-018-4515-2 (PMC5813380; doi:10.1186/s12864-018-4515-2)
Supplement: Supplementary file 7 — Validation by RT-qPCR of the differential expression of mRNA isoforms corresponding to the RXRG, SCD, MAFF and ITGA5 genes in HIGH vs LOW pigs. (PPT 132 kb) [file 12864_2018_4515_MOESM7_ESM.ppt]

## Slide 1
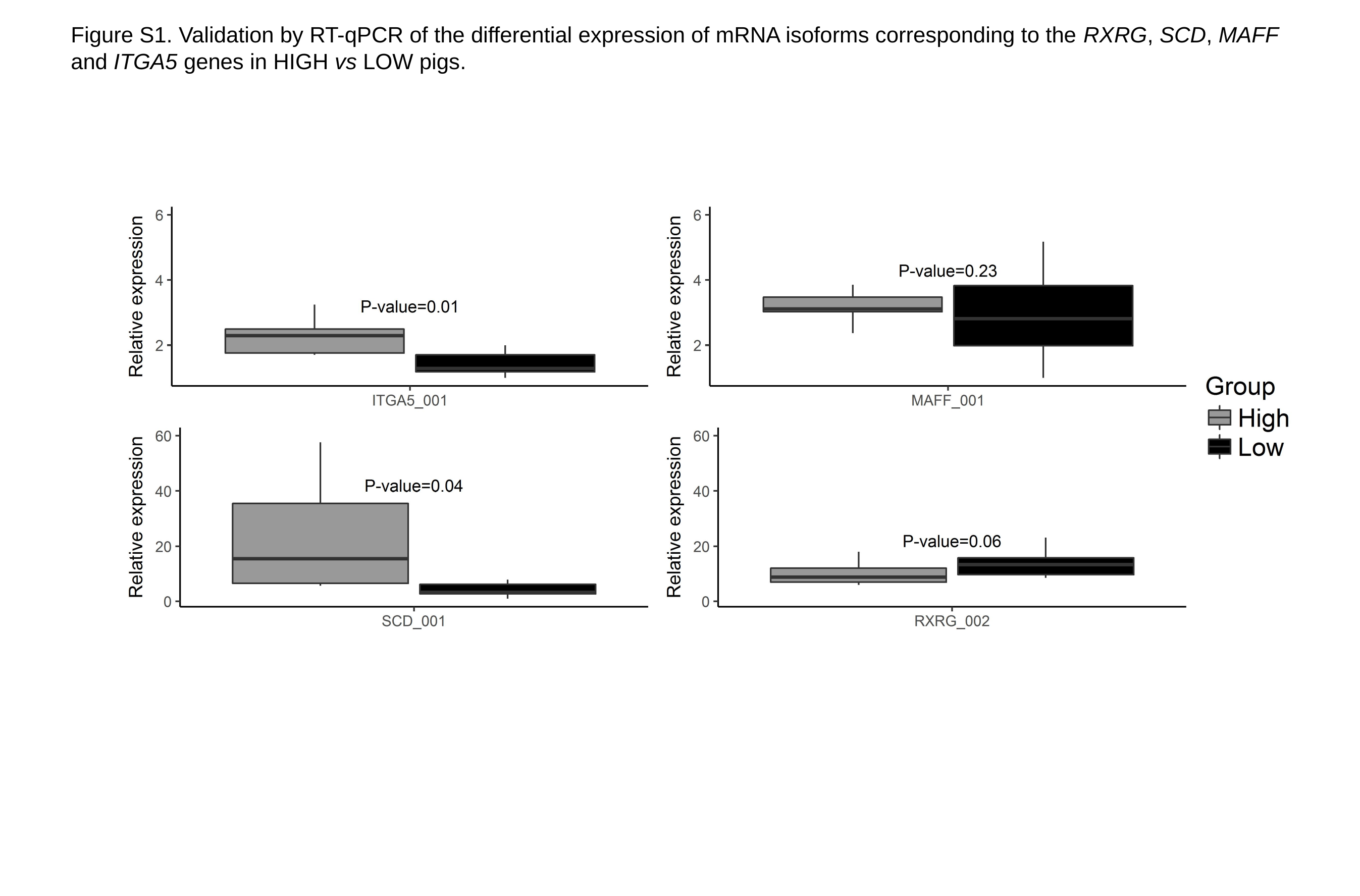

Figure S1. Validation by RT-qPCR of the differential expression of mRNA isoforms corresponding to the RXRG, SCD, MAFF and ITGA5 genes in HIGH vs LOW pigs.
